# Assessment of sociodemographic factors associated with time to self-reported COVID-19 infection among a large multi-center prospective cohort population in the southeastern United States

**DOI:** 10.1371/journal.pone.0293787

**Published:** 2024-09-06

**Authors:** Andrew J. Beron, Joshua O. Yukich, Andrea A. Berry, Adolfo Correa, Joseph Keating, Matthew Bott, Thomas F. Wierzba, William S. Weintraub, DeAnna J. Friedman-Klabanoff, Morgana Mongraw-Chaffin, Michael A. Gibbs, Yhenneko J. Taylor, Patricia J. Kissinger, Devin V. Hayes, John S. Schieffelin, Brian K. Burke, Richard A. Oberhelman

**Affiliations:** 1 Department of Tropical Medicine and Infectious Diseases, Tulane School of Public Health and Tropical Medicine, New Orleans, Louisiana, United States of America; 2 Center for Vaccine Development and Global Health and Department of Pediatrics, University of Maryland School of Medicine, Baltimore, Maryland, United States of America; 3 Department of Population Health Science, University of Mississippi Medical Center, Jackson, Mississippi, United States of America; 4 Milken Institute School of Public Health, The George Washington University, Washington, District of Columbia, United States of America; 5 Section of Infectious Diseases Department of Internal Medicine, Wake Forest University School of Medicine, Winston-Salem, North Carolina, United States of America; 6 MedStar Health Research Institute, Hyattsville, Maryland, United States of America; 7 Georgetown University, Washington, District of Columbia, United States of America; 8 Department of Epidemiology and Prevention, Wake Forest University School of Medicine, Winston-Salem, North Carolina, United States of America; 9 Department of Emergency Medicine, Atrium Health, Charlotte, North Carolina, United States of America; 10 Center for Health System Sciences, Atrium Health, Charlotte, North Carolina, United States of America; 11 Department of Epidemiology, Tulane School of Public Health and Tropical Medicine, New Orleans, Louisiana, United States of America; 12 Vysnova Partners, Landover, Maryland, United States of America; 13 Department of Pediatrics, Tulane School of Medicine, New Orleans, Louisiana, United States of America; Kyung Hee University School of Medicine, REPUBLIC OF KOREA

## Abstract

**Objective:**

We aimed to investigate sociodemographic factors associated with self-reported COVID-19 infection.

**Methods:**

The study population was a prospective multicenter cohort of adult volunteers recruited from healthcare systems located in the mid-Atlantic and southern United States. Between April 2020 and October 2021, participants completed daily online questionnaires about symptoms, exposures, and risk behaviors related to COVID-19, including self-reports of positive SARS CoV-2 detection tests and COVID-19 vaccination. Analysis of time from study enrollment to self-reported COVID-19 infection used a time-varying mixed effects Cox-proportional hazards framework.

**Results:**

Overall, 1,603 of 27,214 study participants (5.9%) reported a positive COVID-19 test during the study period. The adjusted hazard ratio demonstrated lower risk for women, those with a graduate level degree, and smokers. A higher risk was observed for healthcare workers, those aged 18–34, those in rural areas, those from households where a member attends school or interacts with the public, and those who visited a health provider in the last year.

**Conclusions:**

We identified subgroups within healthcare network populations defined by age, occupational exposure, and rural location reporting higher than average rates of COVID-19 infection for our surveillance population. These subgroups should be monitored closely in future epidemics of respiratory viral diseases.

## Introduction

The COVID-19 pandemic has created and widened existing health disparities within US society. Epidemiologic data collected during COVID-19 surveillance provides important insights on at-risk populations including those with differential access to information and infection control measures, in particular social distancing, masking, and new vaccines. Regional studies carried out by local U.S. healthcare and public health organizations, as well as national cross-sectional studies [1–3], have found a higher incidence of COVID-19 illness in ethnic and race-based minority populations and certain age groups [4]. Nonetheless, there is a paucity of comprehensive risk factor analyses beyond demographic characteristics that examine other factors such as occupation, self-reported health conditions, health behaviors, and household characteristics.

The COVID-19 Community Research Partnership (CCRP) was a multi-state cohort study designed to monitor the evolution of the pandemic in a large population with both syndromic surveillance and periodic testing for serologic evidence of infection. The goal of the CCRP was to generate data to inform ongoing public health responses to COVID-19 as well as future pandemics by recruiting a diverse cohort of patients and community members. The methods and purpose have been described elsewhere [5].

This large, multicenter study provided an opportunity to further examine population-based risk factors for COVID-19 to identify characteristics of subgroups at highest risk for becoming infected. As such, the purpose of this study is to investigate factors associated with time from enrollment in the study to a self-reported COVID-19 infection. Herein, we report findings of our risk factor analysis comparing hazard rates in subgroups based on individual and household characteristics.

## Materials and methods

Participants were members of a prospective, multi-site, CCRP COVID-19 surveillance cohort study, a convenience sample of patients and healthcare workers in ten healthcare systems from the Mid-Atlantic and southeastern US. We recruited adults through patient portals or email from the following health systems and institutions: Wake Forest Baptist Health, Atrium Health, Wake Med, New Hanover Regional Medical Center, Vidant Health, Campbell University, Tulane University affiliated partner systems, University of Mississippi, University of Maryland Medical System, and Medstar Health. Adults aged 18 years and older were eligible to participate if they were a patient or employee of a participating healthcare system. Only participants who reported residing in Maryland, Virginia, Washington D.C., North Carolina, South Carolina, Mississippi, and Louisiana were included. Participants who reported a prior positive test for COVID-19 on enrollment were excluded.

Study procedures included daily online questionnaires for all participants, extracted information from electronic health records for those who were patients at a participating health system, and periodic at-home serological testing for a subset of participants. The Wake Forest Baptist Health Institutional Review Board (IRB), which served as the central IRB for this study, approved the study protocol. Internet-based informed consent through secure patient portals was obtained from study participants prior to any study procedures. The study is registered with ClinicalTrials.gov, NCT04342884. Enrollment began April 8, 2020, and ended on April 29, 2022.

### Participant data

Participants in the study completed daily online questionnaires about symptoms, exposures, risk avoidance behaviors related to COVID-19, and self-reported any recent positive SARS CoV-2 detection tests (herein described as “self-reported COVID-19”). Respondents also reported their history of COVID-19 vaccination (date of receipt, product, dose 1 or dose 2, participation in a clinical trial). Race was defined based on responses to the initial study enrollment questionnaire, with options listed as 1) Black or African American, 2) Asian, 3) Hispanic or Latino, 4) White (not Hispanic/Latino), 5) American Indian or Alaskan Native, and 6) Mixed Ethnicity. Participants were invited to complete two supplemental online questionnaires, one that was focused on the individual and another that was focused on the individual’s household, to provide more detailed information on demographic characteristics, occupations, self-reported health conditions, health behaviors, and household characteristics. Supplemental questionnaires were sent to all actively enrolled participants in May 2021, and subsequent newly enrolled study participants received the surveys within one month of starting the study.

We examined correlations between the occupational characteristics of participants and the primary outcome. The National Institute for Occupational Safety and Health (NIOSH), a United States federal agency responsible for conducting research and making recommendations for the prevention of work-related injury and illness, has characterized workplace exposure to SARS-CoV-2 in hundreds of non-health care occupations using metrics from O*NET, a national database with information on occupational characteristics, together with input from experts in occupational safety and health. Based on these data, occupations are categorized in three risk levels (i.e., high, medium, low) using the SARS-CoV-2 Occupational Exposure Matrix (SOEM) system [6]. For the purposes of our study, SOEM exposure categories were based on three factors identified as contributing to increased risk of exposure in the workplace: whether an occupation involves routine in-person interaction with the public (*Public Facing*), working indoors (*Working Indoors*), and working in close physical proximity to others, either co-workers or the public (*Close Proximity*). Since health care workers were over-represented in the study population, the high exposure group was divided into two groups for data analysis, i.e., high-healthcare, and high-non healthcare.

### Data analysis

Data from participants who completed both supplemental questionnaires were analyzed to determine demographic, occupational, health-related, and behavioral correlates of self-reported SARS CoV-2 infection. The analysis was done in R Version 4.3.3. Using the RStudio Desktop User Interface and relied on the following additional packages: ‘survival’, ‘survminer,’ and ‘coxme.’ P <0.05 defined statistical significance. Descriptive statistics were produced by cross tabulation and covariates for model inclusion were first checked for collinearity using pairwise correlation. Analysis followed a time-varying mixed effects Cox-proportional hazards framework, with a shared frailty at the level of recruitment site to account for homogeneity within each site/health network and to account for intraclass correlation in the outcome of interest (self-reported COVID-19 infection) within health networks. Hazard Ratios and 95% confidence intervals are reported as unadjusted estimates and as adjusted estimates for all covariates included in the final model (Table 2). Survival-curves are also presented using the Kaplan-Meier Product Limit estimator for selected covariates.

Two time-varying covariates were included in the categorical hazard analysis: 1) daily county level 7-day average COVID-19 incidence data published by the New York Times in 2020 and 2021 [7]. and 2) COVID-19 vaccination status of participants. Participants were considered vaccinated after they reported receiving their first vaccination dose of any COVID-19 vaccine.

## Results

A total of 69,714 participants were enrolled in the CCRP study. Of those, 42,701 (61.3%) completed the individual adult supplemental survey and 31,642 (45.4%) completed the household supplemental survey. Just under thirty thousand (29,973 [43.0%]) participants completed both the individual adult and household supplemental surveys. Participants that reported residing outside of Maryland, Virginia, Washington D.C., North Carolina, South Carolina, Mississippi, and Louisiana, as well as participants that were participating in clinical trials were excluded, leaving a total of 27,214 participants (39% of the total number of CCRP study participants) in the analytic population. The median follow-up time was 307 days with an interquartile range of 246 days (255–501). Self-reported COVID 19 was considered an event and all other survey responses were considered right censored. The earliest start date in the data is 2020–04–09 and the last follow up date is 2021–10–31. The shortest amount of follow-up time for a single participant was one day and the longest was 570 days.

Overall, 1,603 of a total of the 27,214 study participants (5.9%) reported that they had a positive test for SARS-CoV-2 between enrollment and the end of October 2021 (Table 1). The study population was predominantly female (71.4%) and White/non-Hispanic (88.3%; Table 1). Most participants lived in counties classified as urban (56.1%) and 96.9% had at least some college education with a large proportion holding graduate level degrees (47.9%). The study population was affluent (54.1% had a household income over $100,000). Data was not available for SOEM category designation for 32% of subjects, but the other participants were evenly distributed between the four SOEM exposure groups (low, medium, high-non healthcare, and high-healthcare). The networks with the largest numbers of participants included Wake Forest (32.6%), MedStar (26.7%), and Atrium (17.1%).

**Table 1:** Characteristics at baseline of participants enrolled in the COVID-19 Community Research Panel included for analysis by self-reported COVID-19 status.

Variable	Overall number of participants*	No*	Yes*	Percentage with Self-Reported COVID-19 Diagnosis
Total subjects	27,214	25,611	1,603	5.9
Female	19,443 (71.4)	18,266 (71.3)	1,177 (73.4)	6.1
Age group				
18–34	2,911 (10.7)	2,675 (10.4)	236 (14.7)	8.1
35–49	7,395 (27.2)	6,808 (26.6)	587 (36.6)	7.9
50–64	9,388 (34.5)	8,832 (34.5)	556 (34.7)	5.9
65+	7,520 (27.6)	7,296 (28.5)	224 (14.0)	3
Race/Ethnicity				
White NH	24,032 (88.3)	22,582 (88.2)	1,450 (90.5)	6
Black NH	1,439 (5.3)	1,377 (5.4)	62 (3.9)	4.3
Hispanic	604 (2.2)	563 (2.2)	41 (2.6)	6.8
Asian	488 (1.8)	470 (1.8)	18 (1.1)	3.7
Other	651 (2.4)	619 (2.4)	32 (2.0)	4.9
County Classification				
Rural	5,587 (20.5)	5,184 (20.2)	403 (25.1)	7.2
Suburban	6,355 (23.4)	5,950 (23.2)	405 (25.3)	6.4
Urban	15,271 (56.1)	14,476 (56.5)	795 (49.6)	5.2
Health Care Worker lives in Household	6,006 (22.1)	5,441 (21.2)	565 (35.2)	9.4
Education Level of Respondent				
Hs or less	845 (3.1)	782 (3.1)	63 (3.9)	7.5
Some college	2,756 (10.1)	2,564 (10.0)	192 (12.0)	7
Associate	2,090 (7.7)	1,882 (7.3)	208 (13.0)	10
Bachelor	8,483 (31.2)	7,927 (31.0)	556 (34.7)	6.6
Graduate	13,040 (47.9)	12,456 (48.6)	584 (36.4)	4.5
History of Influenza Vaccination	26,338 (96.8)	24,800 (96.8)	1,538 (95.9)	5.8
Time Since Last Primary Care Visit				
<1 Year	22,590 (83.0)	21,232 (82.9)	1,358 (84.7)	6
1–2 Years Ago	3,311 (12.2)	3,137 (12.2)	174 (10.9)	5.3
2–5 Years Ago	912 (3.4)	871 (3.4)	41 (2.6)	4.5
>5 Years Ago	401 (1.5)	371 (1.4)	30 (1.9)	7.5
Current Tobacco Use of Respondent	1,485 (5.5)	1,410 (5.5)	75 (4.7)	5.1
Number of Concurrent Medical Conditions Reported				
None	9,073 (33.3)	8,520 (33.3)	553 (34.5)	6.1
One	8,961 (32.9)	8,431 (32.9)	530 (33.1)	5.9
Two	5,603 (20.6)	5,294 (20.7)	309 (19.3)	5.5
3 or More	3,577 (13.1)	3,366 (13.1)	211 (13.2)	5.9
Household Annual Income				
<50k	2,749 (10.1)	2,595 (10.1)	154 (9.6)	5.6
50-100k	7,061 (25.9)	6,559 (25.6)	502 (31.3)	7.1
>100k	14,732 (54.1)	13,924 (54.4)	808 (50.4)	5.5
No answer	2,672 (9.8)	2,533 (9.9)	139 (8.7)	5.2
Someone in the Household Attends Class in Person	6,911 (25.4)	6,315 (24.7)	596 (37.2)	8.6
Social Contant Worker in the Household	10,279 (37.8)	9,471 (37.0)	808 (50.4)	7.9
Study Site				
Atrium	4,662 (17.1)	4,221 (16.5)	441 (27.5)	9.5
Campbell	247 (0.9)	238 (0.9)	9 (0.6)	3.6
Maryland	3,366 (12.4)	3,298 (12.9)	68 (4.2)	2
MedStar	7,265 (26.7)	7,100 (27.7)	165 (10.3)	2.3
Mississippi	182 (0.7)	172 (0.7)	10 (0.6)	5.5
New Hanover	438 (1.6)	408 (1.6)	30 (1.9)	6.8
Tulane	79 (0.3)	71 (0.3)	8 (0.5)	10.1
Vidant	619 (2.3)	585 (2.3)	34 (2.1)	5.5
Wake Forest	8,883 (32.6)	8,132 (31.8)	751 (46.8)	8.5
Wake Med	1,473 (5.4)	1,386 (5.4)	87 (5.4)	5.9
SOEM Exposure Risk Categorization of Subjects Occupation				
High—Healthcare	5,080 (20.1)	4,587 (19.4)	493 (31.4)	9.7
High—Other	5,179 (20.5)	4,849 (20.5)	330 (21.0)	6.4
Low	4,316 (17.1)	4,068 (17.2)	248 (15.8)	5.7
Medium	2,100 (8.3)	1,948 (8.2)	152 (9.7)	7.2
No Data	8,577 (34.0)	8,231 (34.8)	346 (22.1)	4

* Values are all n (%) unless otherwise noted. No = Did not report COVID-19 infection during surveillance period; Yes = Did report COVID-19 infection during surveillance period

Adjusted and unadjusted hazard ratios for self-reported COVID-19 are shown in Table 2. Even though a higher proportion of women in the study population reported SARS-CoV-2 infections as compared to men (6.1% vs. 5.5%), the adjusted hazard ratio demonstrated lower risk for women (aHR = 0.87 [0.77–0.98]). When compared to the risk of self-reported COVID-19 among participants ages 18–34, all other age groups had a significantly lower risk for infection (vs. ages 35–49 aHR = 0.80; vs. ages 50–64 aHR = 0.63; vs. ages >65 aHR = 0.45). No difference in risk of COVID-19 was seen based on race or ethnicity.

**Table 2:** Determinants of self-reported COVID-19 diagnosis following a time-varying mixed-effects Cox-proportional hazards model with shared frailty for health system (N = 27,214).

Term	Unadjusted Hazard Ratio (95% CI)	Adjusted Hazard Ratio (95% CI)
**Sex**		
Male	1.0	1.0
Female	1.07 (0.96–1.19)	0.88 (0.78–0.99)*
**Age**		
Age 18–34	1.0	1.0
Age 35–49	0.93 (0.79–1.09)	0.79 (0.67–0.92) *
Age 50–64	0.76 (0.62–0.94) *	0.61 (0.52–0.71) *
Age 65+	0.69 (0.56–0.85) *	0.44 (0.35–0.54) *
**Race-Ethnicity**		
Non-Hispanic White	1.0	1.0
Non-Hispanic Black	0.88 (0.68–1.14)	0.85 (0.66–1.10)
Hispanic	1.31 (0.96–1.79)	1.11 (0.81–1.51)
Asian	0.71 (0.45–1.13)	0.79 (0.49–1.25)
Other Race/Ethnicity	1.00 (0.71–1.42)	0.85 (0.59–1.21)
**Urban-Rural**		
County Rural	1.0	1.0
County Suburban	0.92 (0.80–1.06)	0.97 (0.84–1.12)
County Urban	0.66 (0.58–0.75) *	0.88 (0.78–1.01)
**Education**		
High School or Less	1.0	1.0
Some college	0.94 (0.71–1.25)	0.85 (0.64–1.14)
Associate Degree	1.21 (0.91–1.60)	0.95 (0.72–1.27)
Bachelor Degree	0.87 (0.67–1.13)	0.78 (0.59–1.02)
Graduate Degree	0.64 (0.49–0.83)	0.57 (0.44–0.76) *
**Influenza Vaccine History**		
Never Had Influenza Vaccine	1.0	1.0
Ever Had Influenza Vaccine	0.78 (0.61–1.00)	0.93 (0.72–1.20)
**Time Since Last Primary Care Visit**		
Last Primary Care Visit < 1 Year Ago	1.0	1.0
Last Primary Care Visit 1–2 Years Ago	0.94 (0.80–1.10)	0.84 (0.71–0.98) *
Last Primary Care Visit 2–5 Years Ago	0.84 (0.62–1.15)	0.68 (0.49–0.94) *
Last Primary Care Visit >5 Years Ago	1.32 (0.92–1.89)	1.10 (0.77–1.59)
**Tobacco Use**		
Not A Current Tobacco User	1.0	1.0
Current Tobacco User	0.88 (0.70–1.11)	0.73 (0.58–0.92) *
**Co-morbidities**		
No Concurrent Medical Conditions	1.0	1.0
One Concurrent Medical Condition	0.98 (0.87–1.10)	1.01 (0.90–1.14)
Two Concurrent Medical Conditions	0.91 (0.79–1.04)	1.01 (0.88–1.17)
3 or More Concurrent Medical Conditions	0.99 (0.85–1.16)	1.18 (0.99–1.40)
**Household Income**		
Household Income <50k	1.0	1.0
Household income 50-100k	1.27 (1.06–1.52) *	1.22 (1.01–1.47) *
Household income >100k	1.11 (0.93–1.32)	1.14 (0.94–1.38)
Household income No answer	0.96 (0.76–1.21)	1.04 (0.82–1.33)
**In-person class**		
No one in Household attends in person class	1.0	1.0
Someone in Household attends in person class	1.64 (1.48–1.82) *	1.28 (1.14–1.43) *
**Social Contact Occupation**		
No one in Household works in a Social Contact Occupation	1.0	1.0
Someone in Household works in a Social Contact Occupation	1.60 (1.45–1.77) *	1.24 (1.12–1.38) *
**COVID-19 Vaccination**		
Has not yet had any COVID-19 Vaccine Doses	1.0	1.0
Had At Least One Vaccine Dose	0.26 (0.23–0.30) *	0.37 (0.32–0.42) *
**SOEM Risk Class**		
SOEM—Class High Health Care Work	1.0	1.0
SOEM—Class High	0.85 (0.74–0.98) *	0.80 (0.69–0.93) *
SOEM Class Medium	0.94 (0.78–1.13) *	0.80 (0.66–0.97) *
SOEM—Class Low	0.81 (0.69–0.94) *	0.74 (0.63–0.87) *
SOEM Class No Data	0.47 (0.41–0.54) *	0.58 (0.49–0.68) *

* p ≤ 0.05; Adjusted HR are adjusted for Sex, Age, Race-Ethnicity, Urban-Rural, Education, Influenza Vaccine History, Time Since Last Primary Care Visit, Tobacco Use, Co-morbidities, Household Income, In-person class, Social Contact Occupation, COVID-19 Vaccination and SOME Risk Class.

**Fig 1:**
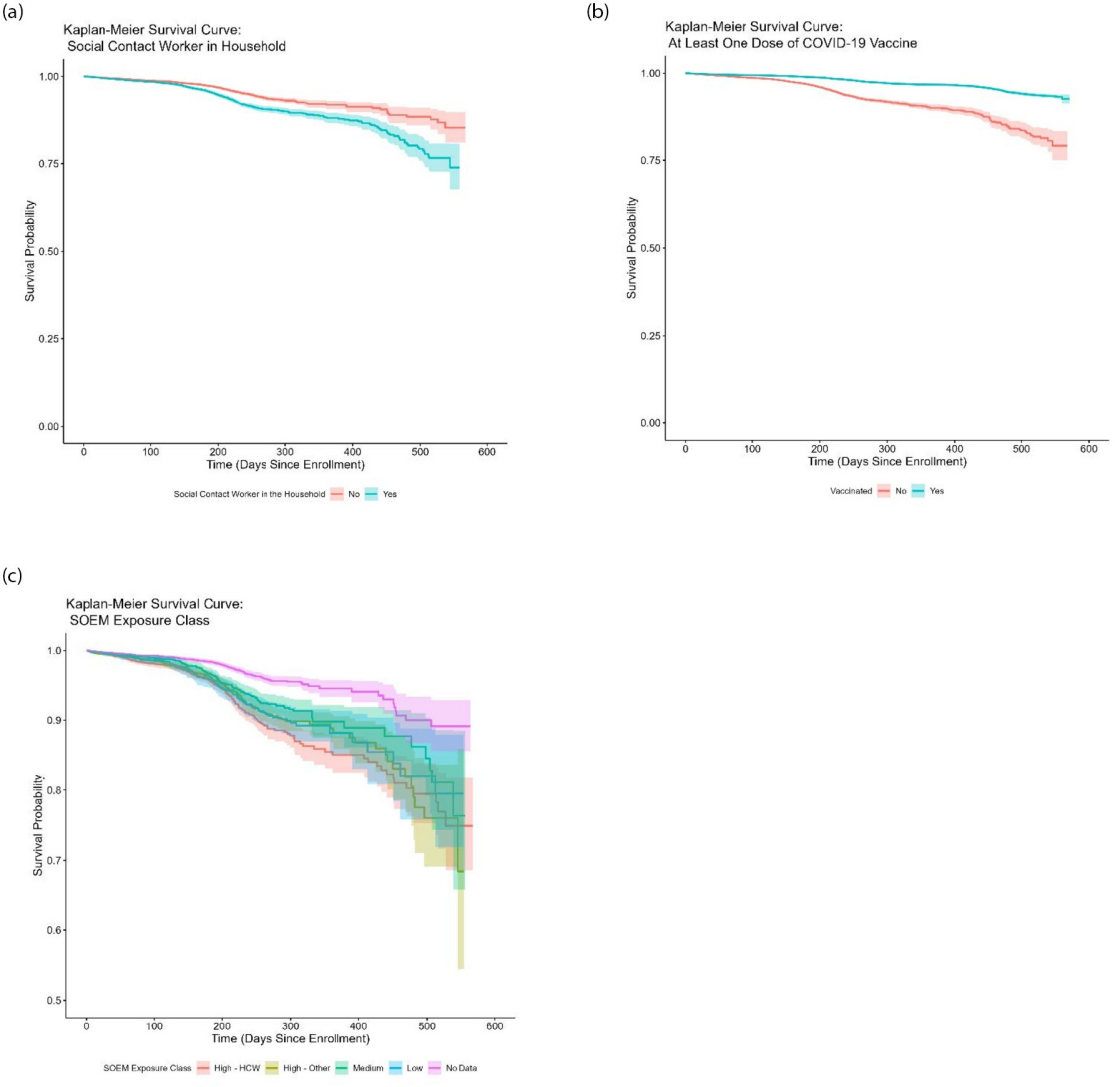


In unadjusted analysis, participants living in urban counties had a lower risk of self-reported infection compared to those living in rural counties (HR 0.66 [0.58–0.75]), but this was attenuated and only borderline significant in adjusted analysis (aHR = 0.88 [0.77–1.00]). Educational level was strongly associated with risk, with a significantly lower risk for participants with a graduate level degree (aHR = 0.57 [0.44–0.76]) compared to those with no college education. As compared to participants who had seen their primary care provider within the past year, a lower risk was observed for those whose last primary care visit was 1–2 years ago (aHR = 0.84 [0.71–0.98]) or 2–5 years ago (aHR = 0.68 [0.49–0.94]). Smokers had significantly lower risk of infection as compared to non-smokers (aHR = 0.73 [0.58–0.92]). Risk did not correlate with the number of self-reported health conditions, but there was a borderline significant increase in risk for participants with 3 or more chronic health conditions as compared to those who reported no chronic conditions (aHR = 1.18 [0.99–1.40]). Household income was not significantly associated with risk. Higher risk was observed in households where someone attended classes in-person (aHR = 1.23 [1.11–1.37]) and in households where someone had occupational contact with the general public (aHR = 1.24 [1.12–1.38]; Fig 1a). Receipt of at least one dose of COVID-19 vaccine was strongly protective (aHR = 0.37; Fig 1b). In adjusted analyses based on SOEM occupational risk level, the high-risk/healthcare group had a higher risk as compared to the high risk/non healthcare group (aHR = 0.79 [0.68–0.92]), the medium risk group (aHR = 0.82 [0.68–1.00]), and the low-risk group (aHR = 0.75 [0.64–0.89]; Fig 1c).

## Discussion

Our multicenter COVID-19 Community Research Partnership study provided a unique opportunity to examine the effect of demographic factors on the risk of SARS CoV-2 infections in populations across the southern United States. After adjustment, we found that multiple social and economic factors were strongly associated with self-reported SARS CoV-2 infection during the pandemic. Risk of infection was significantly higher in young adult participants ages 18–34 years as compared to older groups, and the hazard ratios indicated that risk of infection as compared to the youngest participant group decreased further with each sequentially older age stratum, which is consistent with findings from other studies [8, 9]. This observation may be related to increasing concern about disease outcome in older age groups leading to greater adoption of preventative behaviors among older age cohorts. In contrast to the findings from other studies [10–12], we found no association between race or ethnicity and the risk of self-reported infection, a finding that may reflect the underrepresentation of minorities and/or the relative affluence of our study population. Participants with graduate level college education also demonstrated lower risk as compared to participants without college education, again consistent with other studies [13]. Potential explanations for this observation include a lower likelihood of work-related contact with the general public and a greater awareness of risk and effective methods of protection among more educated subjects. The observation of lower risk among those whose last encounter with a primary care provider 1–5 years ago as compared to those who saw their provider within the past year could be due to greater awareness of COVID-19 from the health care provider, or to health care visits related to known or suspected COVID-19 illnesses.

Counterintuitively, smokers appeared to have a lower risk of infection as compared to non-smokers. This paradoxical relationship between smoking and risk of illness from COVID-19 has been observed in several other studies [14, 15], including some that speculate that nicotine could mitigate the effect of the cytokine storm in COVID-19 patients [16]. However, these studies also point out that smokers are underrepresented in many COVID-19 population-based studies, as they appear to be in our study as well (5.4% of participants vs. the CDC estimated average of 12.4% for the southern United States in 2021) [17]. Overall, the harmful effects of smoking are often cited as offsetting any possible benefit from smoking for COVID-19 related morbidity.

As expected, participants with a higher occupational risk of exposure to the general public demonstrated a coincident higher risk of infection, including those living in a household with an individual who attends in-person classes or who encounters the general public in their workplace. When risk was compared by occupational group using the NIOSH SOEM risk categories, the most significant increase in risk was from working in a healthcare setting. These results may be used to inform identification of high-risk groups for future respiratory disease outbreaks, allowing targeted programs to promote protective measures and behaviors that reduce the risk of infection.

Our study is subject to several limitations. COVID-19 cases were ascertained based on self-reported data from daily surveys, so the infections could not be independently verified. Participants varied in the frequency with which they responded to the daily surveys, which limits our ability to determine the exact date of any reported case of COVID-19. Generalizability is also limited due to the overrepresentation of lower risk socioeconomic groups in the study population and to recruitment that was limited to patients from healthcare networks and healthcare workers. As mentioned in the results section, this analysis was also limited by the percentage of participants (39%) who completed the supplemental questionnaires.

In contrast, the strengths of this study include a large sample size from a wide geographic area enrolled early in the pandemic and surveyed for a number of demographic, social, and economic characteristics. Our study population was recruited from 7 large health systems or networks that provide care through affiliated hospitals, clinics, and physicians in urban areas. This is the most common model for healthcare in the United States, where 69.7% of hospitals and 42.7% of physicians are in health systems, and 91.6% of hospital discharges are from system hospitals [18]. While there are no data about the racial or age distribution of US adults covered by health systems versus. those who are not, data are available demonstrating that racial minorities are overrepresented among US adults without health insurance (in 2022, 20.9% of Hispanic adults, 10.4% of non-Hispanic blacks, and 6.4% of non-Hispanic whites were uninsured) [19]. As our population was composed of individuals with health insurance from several networks in 5 states and the District of Columbia, we believe that it is similar to the populations of other US health networks, and our results are informative for identifying characteristics which can help in future pandemics for other US health system populations.

Our results validate findings from other studies and expand the body of evidence with several novel features. The categorical hazard analysis in this diverse study population is strengthened by employing two time-varying covariates: 1) county level 7-day average COVID-19 incidence data updated daily and published by the New York Times in 2020 and 2021 and 2) COVID-19 vaccination status of participants. The study also examined correlations between the occupational characteristics of participants and self-reported COVID-19 using metrics from O*NET, a national database with information on occupational characteristics developed by the National Institute for Occupational Safety and Health (NIOSH), categorized using the SARS-CoV-2 Occupational Exposure Matrix (SOEM) system. These results have important public health significance due to the size of the multicenter study population, the rigorous analytical approach, and the use of novel categorical metrics such as the SOEM system. Specifically, our results suggested a higher risk for men, those who do not have with a graduate level degree, healthcare workers, younger adults aged 18–34, those in rural areas, those from households where a member attends school or interacts with the public—all groups that are likely to be at higher risk in future pandemics and appropriate targets for increased efforts to prevent infections.

While our results illustrate the challenges inherent in understanding risk based on the complex interaction of age, race, and occupation and disentangling this from the risk due to local community infection rates, they also provide potential target groups for ongoing intervention. Under the assumption that those most at risk for first SARS-CoV-2 infection remain at higher risk for repeated infections, even in the changing risk environment from vaccine uptake and shifting perceptions of the necessity of mitigations, this study may provide insight into ways to reduce ongoing disparities from the pandemic through risk stratification and targeted interventions.

## Abbreviations

CCRP: COVID-19 Community Research Partnership
CDC: Centers for Disease Control and Prevention
IRB: Institutional Review Board
